# Mindfulness-Based Cognitive Approach for Seniors (MBCAS): Program Development and Implementation

**DOI:** 10.1007/s12671-013-0262-2

**Published:** 2013-11-28

**Authors:** Brigitte Zellner Keller, Nirbhay N. Singh, Alan S. W. Winton

**Affiliations:** 1 Institute of Psychology, Gerontology, University of Lausanne, Bat. Géopolis, 1015 Lausanne, Switzerland; 2 Department of Psychiatry and Health Behavior, Medical College of Georgia, Georgia Regents University, Augusta, GA USA; 3 School of Psychology, Massey University, Palmerston North, New Zealand

**Keywords:** Aging and self-development, MBCAS, Mindfulness group for seniors, Mindfulness and memories

## Abstract

A number of cognitive interventions have been developed to enhance cognitive functioning in the growing population of the elderly. We describe the Mindfulness-Based Cognitive Approach for Seniors (MBCAS), a new training program designed especially for seniors. It was conceived in the context of self-development for seniors who wish to enhance their relationship with their inner and outer selves in order to navigate their aging process more easily and fluently. Physical and psychosocial problems related to aging, as well as some temporal issues, were taken into account in developing this program. Unlike clinically oriented mindfulness-based programs, which are generally delivered during an 8-week period, the MBCAS training program is presented over a period of 8 months. The main objectives of this program are to teach seniors to observe current experiences with nonjudgmental awareness, to identify automatic behaviors or reactions to current experiences that are potentially nonadaptive, and to enhance and reinforce positive coping with typical difficulties that they face in their daily lives. Details of the program development and initial implementation are presented, with suggestions for evaluating the program's effectiveness.

## Introduction

The world's population is aging, with more elderly people alive than ever before, and their proportion of the world's population is increasing. For example, in Switzerland during 1990 to 2011, the proportion of people over 65 rose from 5.8 to 17.2 % and those over 79 from 0.5 to 4.8 % (Swiss Federal Statistical Office 2013), while in the USA during 2000 to 2011, the proportion of people over 65 increased 18 % (Administration on Aging 2012). There are several reasons for population aging, including increased longevity, declining fertility, and aging of “baby boom” generations.

Aging is a dynamic process that can introduce erratic disturbances to the flow of life. It is often accompanied by declines in cognition, physical health, and functional ability (Li et al. 2007; Smith et al. 2002). Numerous physical, cognitive, and social changes take place, such as recurrent redefinitions of healthy balance and various psychosocial and affective events along with personal development imperatives (Blackburn and Dulmus 2007). However, advancing age is also conducive to the integration of life experiences (Krause 2009) and is a fertile period for attributing meaning. While this can be a source of anxiety and negative thoughts, the spiritual life often becomes more important. Aging is also a period of life where a historical repertoire of habits and routines are automatically activated in response to most of these troubles (Baltes et al. 2006). The problem is that these automatic behavioral and cognitive reactions are not always efficient for facing some situations. Yet they persist as they belong to the psychological patterns of a person—patterns that have been selected, refined, and reinforced throughout life. Fortunately, lifestyle changes can slow age-related declines, often dramatically (Clark et al. 2012; Hendricks and Hatch 2009).

The concept of cognitive and neural plasticity has been used to provide interventions to the elderly to enhance their performance in multiple areas (Ball et al. 2002). Life span theories are aligned with the concept of plasticity of brain functions in terms of development as a process of lifelong adaptation to aging (Baltes et al. 2006) and to underlie various cognitive remediation strategies. Recently, there has been a growing interest in teaching mindfulness-based practices to the aging population and their caretakers (McBee 2003; Rejeski 2008; Smith 2004). Mindfulness practices enable people to observe and attend to their moment-to-moment experiences, with nonjudgmental awareness. At the heart of this practice is the realization that thoughts arise but they do not exist and, thus, no response to thoughts is required. With consistent practice of mindfulness-based techniques, especially insight meditation, an individual can learn to adaptively regulate his or her emotions with regard to the daily vicissitudes of life events. Although a number of mindfulness-based techniques have been advanced in the research literature, the Mindfulness-Based Stress Reduction program (MBSR; Kabat-Zinn 1990) is the most widely used mindfulness program at present.

While the literature on using mindfulness-based techniques with older and aging populations is small, it is growing. For example, Young and Baime (2010) reported a retrospective study of 141 participants, aged 70 years or older, who had previously participated in the Penn Program for Mindfulness course. The participants had received 27 h of instruction over an 8-week period, with a 45-min daily homework practice. The results showed clinically significant reductions in depression and anxiety in their subject sample. Similar MBSR training given to a small sample of caregivers in a pilot study showed reductions in self-reported depression, perceived stress, and burden of care following participation in the 8-week course, with further reductions reported at the 1-month follow-up (Epstein-Lubow et al. 2011). In addition, the caregivers reported enhanced mindful attention and calmness over the course of the study. Furthermore, in a recent small, randomized controlled study, Cresswell et al. (2012) reported statistically significant reductions in loneliness in older adults, following participation in an 8-week MBSR training.

The primary purpose of the present paper is to describe the development of a mindfulness-based cognitive program for seniors: the Mindfulness-Based Cognitive Approach for Seniors (MBCAS). A secondary purpose is to discuss its implementation, some initial findings, and considerations about its effectiveness.

## The MBCAS Program

The MBCAS program is based on the MBSR, Mindfulness-Based Cognitive Therapy (MBCT; Segal et al. 2002) and Mindfulness-Based Relapse Prevention (MBRP; Bowen et al. 2011) programs. It is designed for seniors, defined as people 65 years or older, who are healthy or mostly healthy. Its main objectives are to teach them to (a) observe current experiences with nonjudgmental awareness, (b) identify automatic behaviors or reactions to current experiences that are potentially nonadaptive, and (c) enhance and reinforce positive coping with typical difficulties that they face in their daily lives. An experienced mindfulness instructor teaches the MBCAS program.

### Aims

MBCAS shares the following aims with other mindfulness-based programs, such as MBSR, MBCT, or MBRP: (a) to stimulate awareness of automatic responses; (b) to exercise self-observation of mental and emotional events which trigger automatic reactions; (c) to encourage more adaptive responses, as an alternative to automatic reactions; and (d) to develop acceptance of and compassion toward one's own internal and external events. In addition, MBCAS includes specific objectives for seniors, including (a) to stimulate verbal skills related to the consciousness of inner experiences; (b) to move mindfully in the context of one's physical limitations; (c) to experience feelings of losing control, without nurturing other thoughts such as the risk of losing autonomy; (d) to disentangle oneself from negative thoughts specific to aged people (e.g., “I'm too old to change,” “It's normal to feel sad when you get old”) (Tison and Hautekeete 2001); and (e) to enhance and reinforce positive coping with typical difficulties that seniors have to deal with in their daily life (e.g., health problems with irreversible physical consequences, psychosocial limitations such as being unable to drive, or the death of peers).

### Course Duration

The MBCAS program is provided in eight monthly sessions and seven intermediate practice sessions. This is unlike the other mindfulness-based interventions, which are presented in eight weekly sessions. The rationale and reasons for this unusual duration are the following: (a) the underpinning of the program is self-development of seniors and this takes time—a lot more time, given their age and potential for decreasing cognitive abilities; (b) healthy seniors are generally not eager to follow an intensive 8-week program because there is no clinical urgency; (c) healthy seniors have a need to feel competent and thus are not eager to be closely guided; (d) healthy seniors have a need to exercise and explore new learning within their own rhythm of life and a longer intervention period gives more time to practice alone, with regular guidance and group meetings; (e) a longer intervention period provides more opportunities to engage in meditation practice under “real-life conditions” and permits the ritualization of the practice; and (f) with a more relaxed training pace and an enhanced opportunity to really practice, the instructor becomes less important than the participant's experience.

The temporal design of MBCAS is a monthly 2-h session in a closed group, followed 2 weeks later by a 1-h practice session, in an open group that includes current participants, seniors who previously attended the program, students, and academic colleagues. While the number of group sessions, training hours, and cost are similar to other standard mindfulness-based interventions, the opportunities for individual practice during the MBCAS program are four times greater. It would be interesting to assess whether the regularity of daily practice and attendance at the monthly sessions are affected by this particular temporal design.

### Structure

MBCAS uses group training, a format well suited to seniors. As many seniors live alone at home, group training provides an opportunity for social interaction in the local community. The format enables the acquisition of new behaviors through observational learning and modeling what has worked for other group members (Bandura 1977). Furthermore, it provides opportunities for sharing experiences and additional insights from group members, and the motivation to not only participate consistently but also take the training to the next level.

The mindfulness training was conceived as a group process, where participants and instructors meet regularly to learn and practice. The core of any mindfulness program takes place during these practice sessions, where each participant exercises the connection to his or her inner world at the same time as others practicing in the same room, and sharing with others, moment-by-moment. Thus, each participant could report on his or her unique inner experience, which has been knitted with the shared common moment (e.g., the bird song that some have heard and appreciated, while some have not, or a participant's cough). Moreover, people are generally seated in a circle, which enables subtle and inherent information to pass within the group: “When one sits in a circle with others, everyone is equal and linked… Because everyone is interrelated and derives meaning through the relationship of the circle, each person's vision is transformed as the circle takes form” (Murdock 1990, pp. 180–181). The communication process turns from one to another, with the instructor becoming no more important than any other participant.

This group process provides a unique way of learning about “inter-being,” a major pillar of any mindfulness-based program (Hanh 2009). The concept of inter-being is a concise formulation of the doctrine of dependent co-arising—that there is no independent self and all things arise in dependence upon multiple causes and conditions—as explicated in the *paticcasamuppada-vibhanga Sutt*a (Dalai Lama 1992). This linking process—the inter-being—stimulates relationships with others and goes beyond the duality of “I” and “others.” This non-dual exploration is fundamental in any mindfulness-based intervention.

### Contents

The topics covered in the eight monthly sessions included: (1) Who controls my life: my habits or my consciousness? (2) How does my mind work? (3) Breathing as an anchor to the present moment. (4) Developing awareness of my moods. (5) Dealing with uncomfortable situations. (6) How about my future? (7) What can I do: adapt or resist? and (8) Taking care of myself, day after day.

To develop the participant's mindfulness, two kinds of exercises were used: (a) focusing on something specific during meditation, an excellent training to reduce distraction and loss of focus and to increase concentration, and (b) simply “being,” that is, without selecting or avoiding anything that could emerge during a meditation session, which is standard training for cultivating presence and openness moment-by-moment. The progression of the exercises starts with the very tangible bodily sensations and moves toward the less tangible inner experiences (i.e., the observation of one's thoughts and emotions). The meditation and cognitive exercises in MBCAS are similar to those used in other programs but adapted to the needs of the seniors. At the end of each session, the participants receive audio CDs, written material, and home practice exercises.

The basis of mindfulness practice is to cultivate disidentification from one's experience of internal events. For example, the participants are taught, “I am not what I feel or think,” in order to understand that feeling anxious or having negative ideas at one moment does not mean that they are that inner feeling or thought. They learn to observe their thoughts, feelings, and emotions as transient events that pass through their mind but to not hold on to them as being true or descriptive of them (Allen et al. 2009). To reinforce this decentering process (i.e., not believing all of our thoughts), MBCAS enables the participants to learn to establish new relationships with their thoughts, feelings, and emotions, through various exercises. In order to foster the learning of the decentering process, the MBCAS program includes experimenting with the feeling of losing control. Thus, seniors explore their automatic reactions during short experimental situations, where they meet the fear of losing control. For seniors who have tried to master their moods for years and who worry about aging and loss of autonomy, the perspective of developing a new relationship with themselves is deeply touching and hopeful.

As in the other mindfulness-based programs that stimulate body consciousness, MBCAS incorporates mindful movements in various postures and walking exercises. This component is particularly important in the context of aging and often aching bodies, where movements may be affected by arthritic pains or other physical limitations. Participants learn eight adapted movements, in both sitting and standing positions. The objective is to awaken the consciousness of the upper body by gently mobilizing the joints. These movements may stimulate and possibly strengthen muscle tone as well as balance when in a standing position (upper body mobility, motor coordination of hands, and balance on each foot). The MBCAS program provides the opportunity to be more mindful of upper body tensions and movements, allowing better understanding of one's bodily sensations. The participants are invited to practice all eight movements every day.

The walking exercise permits rediscovering a certain quality of openness to and acceptance of the present moment. Participants are encouraged to explore walking at various speeds in order to rediscover their safe balance. They may also try the walking exercise with a small rice bag on top of their head, to practice the feeling of having or losing control, and cultivate their kinesthetic sense of verticality. Even though it is not an objective per se, in time, practicing conscious verticality stimulates deep musculature and frees the head. This results in an enhanced physical presence that inspires dignity.

## Implementation

Six months prior to implementing the program, a free introductory session was announced in the local newspaper. During this 2-h session, the mindfulness approach and the MBCAS program with the title of each of the eight sessions were presented, along with two typical exercises. At the end of the session, people could complete a form to preregister for the program or they could think about it and contact the instructor later, within a specified deadline. Writing the title and dates of each of the eight sessions on the flyers was a key point for many participants in deciding to enroll in the program, with many commenting, “This program is written for me.”

One month before the program began, all potential participants were individually interviewed and invited to set private goals for the particular aspects of themselves they wanted to be more aware of. These personal goals often give clues about how the participants perceive themselves. During the interview, some informal questions were asked regarding health, aches and pains, and important psychosocial events that the participant might have encountered. The French versions of the following assessment instruments were administered: the Geriatric Depression Scale (Brink et al. 1982), the WHO-5 Well-Being Index (Psychiatric Research Unit 1999), and the Rosenberg Self-Esteem scale (Vallieres and Vallerand 1990). Following the interview, those with suspected psychiatric issues were referred for psychiatric services before deciding about their participation in the program. Each participant was then invited to express his or her commitment to following the 8-month program, including home practice.

To date, four groups of participants have completed the MBCAS program (*N* = 43). On average, participants attended 12.6 of the 15 meetings. The percentage of participants who dropped out was low (i.e., 7 %), and 10 % of enrolled participants had to postpone their training for personal reasons to the next time the MBCAS program was run. Periodically, we monitored the frequency and duration of home practice through a random poll. We found that, on average, participants practiced for about 27 min every 1 to 2 days. This is a level of practice that is slightly higher than the usual 15 to 20 min reported in the literature (Kristeller 2007). Former participants, who attended the 1-h monthly practice sessions in the following year, reported meditating at home more than three times a week, for a mean duration of 20 min. This is also slightly higher than the usual 15 min reported by Kabat-Zinn et al. (1987). While we did not undertake a rigorous evaluation of the MBCAS program, a quasi-experimental single-group, pre- and post-training evaluations using the three assessment instruments, indicated significant positive changes in depression, well-being, and self-esteem.

Participants in the four groups were asked to rate on a 10-point scale how meaningful and useful they considered the program. Most rated the program as very meaningful and useful in their life (mean = 8). Most also reported an improved quality of life and also specific personal outcomes such as joy and satisfaction during the 8 months, traversing periods of low motivation without giving up, feeling of release at the realization that thoughts are just thoughts, better insight into one's automatic pilot, enhanced linking to the present moment, self-dignity, better connection to self, and better acceptance of the process (i.e., understanding that acceptance is not resignation). Some also reported that, during the training period, they acquired new behaviors that they considered to be efficient. These included, “I recover faster from emotional events,” “When I lock the door, I no longer need to check it again,” “I try to do less, but try to complete what I start,” and “I have discovered a way of exploring who I am and I am eager to continue this exploration.”

## Discussion

If a participant is reasonably healthy, there is virtually no age limit to developing mindfulness and to finding the energy to mobilize one's inner resources. The MBCAS program provides a context for developing mindfulness in seniors. As there is no clinical motivation to engage in such a program in an intensive way, it provides a low intensity and relaxed approach to developing mindfulness over an 8-month period. One possible obstacle to this program duration could be that the long intersession intervals might be uncomfortable for instructors who are used to providing this type of training in a medical or psychosocial context over 8 weeks. Nevertheless, it might challenge their ability to let go of the process and to truly rely upon the resources that healthy participants are proud to discover and activate. In our experience, the longer time facilitates the emergence of the true nature of each participant.

The 8-month program appears to be well adapted to healthy seniors who wish to invest in a mindfulness-based approach to life. The temporal aspects of the design are realistic and comfortable, allowing participation in the mindfulness program to go beyond learning tricks to feel better. Many of the aged participants in the MBCAS program had previously tried several things, had attended various self-development seminars, engaged in one or more psychotherapies, and had read numerous self-help books. With MBCAS, most participants expressed appreciation for having time to truly learn and practice different ways of being rather than doing. They had spent a lifetime in the doing mode, and they relished learning how to connect with the present moment (e.g., developing an awareness of all senses), to acknowledge things as they are (e.g., active acceptance of the way things are at the moment, being less goal-driven, or losing any insatiable desire to change things), and acquiring a sense of openness to life (e.g., facing all types of emotions with equanimity).

Many participants experienced “off” periods, during which it was difficult to practice, and “on” periods when it was easier. They had time to test, try and apply the recommended exercises, give up, try again, find their own rhythm, lose, and once more find the link to their breathing. This was an important point to learn. When the program was over, participants were not surprised if they encountered variations in their motivation or obstacles that momentarily prevented them from maintaining their practice. Through the 8-month program, they appreciated having time to think in detail about mindfulness practice and having opportunities to share these experiences within the group. For the participants, the choice to engage in the MBCAS program and do the daily exercises was an important decision as it had to be maintained over 8 months. In itself, this process built self-esteem.

Much socialization took place during the 8-month program. Some developed strong relationships with other participants in the course of the training. Others did not build special relationships but really enjoyed the group activity in sessions spaced over time, without feeling stressed by an obligation to be close to others. Almost all participants reconsidered their relationship to solitude by letting their negative representations of solitude dissolve.

Our preliminary quantitative and qualitative data suggest that the MBCAS program may be successful in enabling seniors to activate more constructive and creative responses to the challenges and difficulties in their daily life and to travel on a path of acceptance of the ups and downs of life itself. They appear to be able to observe and respond to what is going on in their lives in a more mindful manner instead of automatically reacting to it. Research studies of MBCAS should focus on evaluating the effectiveness of the program across a broad spectrum of variables that include life satisfaction, role functioning, and self-rated emotional and physical health.

While mindfulness meditation is essentially an experiential and individual practice, verbal communication within the group is an essential component of the MBCAS program because it assists the participants to make sense of these experiences by intertwining the consciousness of one's inner world with the shared outer world. For example, during a breathing exercise each participant may hear a specific song in a particular way and later, through language, share the experience. However, linguists have shown that language is not transparent and, with aging, communication may be physically impaired due to a number of auditory and visual changes (Zellner Keller 2006). Furthermore, words and meanings vary from one person to another, depending on the particular language, education, culture, activities, and life experiences. In addition, meanings vary with time and experience. For example, representations of the word “consciousness” are obviously different before and after participating in a mindfulness-based intervention. Seniors, certainly in Switzerland, are often poorly trained to identify and name their inner states and moods. With this mindfulness training, they can learn both a method and a new lexicon for exploring their inner world.

Group discussions offered a number of opportunities to clarify this instability and opacity in verbal communication. Time and tools may also have contributed to this learning. The seniors often complained about how hard home writing tasks were. Some even felt bad about having difficulties in writing due to bad school memories or feelings of incompetence. It took time to learn how to put inner experiences in words. The 8-month program favored this particular kind of language acquisition, developing patience and perseverance. Discussions around the flipchart contributed to the sharing of experiences and drawings were often helpful. For example, the use of smiley faces was very useful in the naming of different moods. Finally, suggested readings and booklets were helpful to anchor this ongoing learning process at home.

Seniors in the MBCAS program are generally older than the instructors. The ways in which the seniors connect to past events reflect how they navigate back and forth through their life course. For example, our MBCAS participants were mostly retired, their children were adults, and the parents had an empty nest, and they often believed that their affective relationships had been largely explored. Former obstacles could still be very vivid, while others might have been almost forgotten. Sometimes the core of all these difficulties was roughly the same: a similar problem had been faced many times, at different ages, in different situations. For example, a number of participants reported that they had been marginally depressed most of their adult life. Sometimes, their problems required different resources as they had been exposed to largely different experiences. In other words, each participant arrived with an extensive portfolio of memories, stored strategies, and failures. These materials could resurface during the exercises and the discussions in the MBCAS program, revealing that the relationships with these memories could still be emotionally raw. Learning how to relate to these raw memories in a nonjudgmental manner helped them to achieve equanimity in life.

Indeed, some participants may have felt overwhelmed or troubled by their mental activity, ruminating, and feeling stuck in old memories. The proposed "exit" from this entanglement was the voice of their body: the physical sensations were the interface to inner experiences. In MBCAS, the participants were invited to cultivate a deep-rooted consciousness of those bodily responses.

The core of any mindfulness approach is to train participants to concentrate and listen to their bodily sensations, for physical sensations only occur in the present moment. For seniors, this training is assumed to permit a “rereading of one's past with new glasses,” where the past becomes an interesting and constructive living resource. The past is always explored in the present moment, and any projection of the present on memories is understood as not being “the past.” Thus, exploring past events from the present sensations is expected to stimulate the cognitive training of the disidentification process—the capacity to observe oneself without judgment.

Compared to other mindfulness-based programs, the specifics of MBCAS are mainly qualitative adaptations for seniors. Aging can bring about many disturbances in one's life, and the MBCAS is designed to assist seniors in navigating life in a more fluent manner. The art of fluency consists of making things go smoothly, in an easy and effortless manner (Zellner 1994). Practicing mindfulness and being alive in a mindful way is indeed a sure way of developing fluidity in one's life. Each time we embody fluidity, we probably are mindful. This is why a fluent walker does not fall down.

In summary, the MBCAS appears to be a promising program in the areas of mood, acceptance and adaptation to aging, with a more compassionate attitude toward one's body. Engaging seniors in a mindfulness-based approach to life is a realistic objective. Moreover, reasonably healthy seniors are well suited to embark on an 8-month training program. Studies on the short- and long-term effects of MBCAS for reducing the risk of mental and physical aging diseases should be the next step in the development of this program.
